# Editorial for the Special Issue on Miniaturized Transistors

**DOI:** 10.3390/mi10050300

**Published:** 2019-05-02

**Authors:** Lado Filipovic, Tibor Grasser

**Affiliations:** Institute for Microelectronics, TU Wien, Gußhausstraße 27-29/E360, 1040 Vienna, Austria

Complementary Metal Oxide Semiconductor (CMOS) devices and fabrication techniques have enabled tremendous technological advancements in a short period of time. In recent decades transistor scaling has enabled us to fit into our pockets what would be considered a supercomputer a few decades ago. However, as we approach the physical limits of scaling, the question frequently asked is: What is the future of CMOS? Sustaining increased transistor densities along the path of Moore’s Law has become increasingly challenging with limited power budgets, interconnect bandwidths, and fabrication capabilities. In the last decade alone, transistors have undergone significant design makeovers; from planar transistors of 10 years ago, technological advancements have accelerated to today’s FinFETs, which hardly resemble their bulky ancestors. FinFETs could potentially take us to the 5-nm node, but what comes afterwards? From gate-all-around devices to single electron transistors and two-dimensional semiconductors, a torrent of research is being carried out in order to design the next transistor generation, engineer the optimal materials, improve the fabrication technology, and properly simulate future devices.

There are 13 papers published in this Special Issue, covering recent advances in research aspects related to transistor miniaturization, including process and device simulation as well as novel transistor designs and innovative working principles for future transistor technologies. Two reviews are included in this Special Issue, covering technology computer aided design (TCAD) process and device simulations. To enable high performance TCAD and accelerated simulations, alternative meshing strategies are sought after, another topic addressed herein. High performance TCAD is indispensable for the design of future transistor structures. The sophistication and physical accuracy of the semiconductor models which are used today have reached unprecedented levels, allowing researchers to predict the best candidates for next generation devices without ever stepping foot into a fabrication facility. Using semiconductor TCAD, several authors in this Special Issue have proposed and analyzed different transistor materials and geometries. These include transistors based on ferroelectric materials, those based on 2D semiconductors, semi-floating-gate synaptic transistors (SFGSTs), and drain-engineered InGaN heterostructure tunnel FETs (TFETs). Furthermore, several groups have successfully used TCAD to optimize existing structures including SOI MOSFETs, SiGe tunnel FETs, n-channel MOSFETs, and silicon nanowire (SiNW) transistors, These novel transistor geometries and materials require a complex combination of processing steps, which can lead to significant variation in the geometry and ultimately operation of real-world devices. The variability and variation in advanced three-dimensional devices such as FinFETs and stacked nanowires, as well as future gate-all-around (GAA) structures has been addressed by researchers in this Special Issue. In the following, we summarize the individual contributions in this Special Issue, starting with the two reviews followed by 11 scientific research manuscripts.

Klemenschits et al. [1], in their review, summarize methods used to pattern modern gate stacks, which are no longer a single metal or polysilicon layer, but rather a complex stack of materials which must be carefully patterned to create the gate contact. A review of methods used for topography simulations is given therein, including a discussion on the use of explicit and implicit methods to define surfaces during process simulations. Ultimately, the authors describe the methods which made possible the recent advances in the modeling of gate stack patterning using advanced geometries. Enhanced capabilities of today’s simulators and algorithms which accelerate simulation times have been the backbone for allowing modern TCAD to reach such a sophistication to enable the types of research presented in several papers in this Special Issue. In [2], Gnam et al. describe an algorithm they developed in order to accelerate flux calculations when performing process simulations, an essential component of modern process TCAD. With their method they obtained speedups of up to eight times while keeping surface deviations below 3%, ensuring that the simulations retain the high quality and accuracy expected from TCAD models. In the second review in this Special Issue, thin film transistors are addressed. TFTs have recently shown broad potential in applications from RFID tags, logical calculations, and many more. In order to enable circuit simulations with TFTs, fully physical models are not convenient, which is why compact models are indispensable. Lu et al. [3] provide a review of existing compact models for TFTs with different active layers while paying special attention to surface-potential-based compact models of silicon-based TFTs. Ultimately, the review authors propose models which provide accurate circuit-level performance predictions and RFID circuit designs.

Furthermore, Hueting [4], who analyzed current research into ferroelectric transistors. This research field looks at employing ferroelectric materials to obtain positive feedback in the gate control of a switch. The two device architectures analyzed are the NC-FET and the π-FET. The author showed that while the NC-FET shows better performance in terms of subthreshold swing and on current, the π-FET offers a much higher speed of operation. Ultimately a hybrid solution is proposed using a ferroelectric material with a high piezocoefficient. Chang et al. [5] have studied two-dimensional (2D) field effect transistors (FETs) based on indium selenide (InSe), noting that remote phonon and Fröhlich interaction plays a comparatively major role in determining electron transport in InSe. Cho et al. [6] demonstrate a semi-floating-gate synaptic transistor (SFGST) for energy-efficient hardware-driven neuromorphic systems. The authors utilize a poly-Si semi-floating gate and a SiN charge-trap layer which is charged by a tunneling FET which is embedded between the channel and the drain junction. The design is intended to operate as fast as the human brain with low power consumption and high integration density. Duan et al. [7] propose a drain engineered InGaN heterostructure field effect transistor (DE-HTFET) which uses an additional metal on the drain region to modulate the energy band near the drain/channel interface. Their design showed a reduction in the subthreshold swing by 53.3% and a doubling of I${}_{\mathrm{ON}}$ compared to nonpolar DE-HTFETs.

In order to improve the performance of inversion-channel and buried-channel SOI MOSFETs, Omura [8] looks at their low-frequency noise behavior at sub-100 nm channel widths. The author proposes models which explain why the low-frequency noise in the buried channel MOSFET is primarily influenced by interface traps near the top of the surface of the SOI layer and not the traps near the bottom surface of the SOI. Yang et al. [9] proposed a TFET using SiGe source and drain regions which increase the ESD failure current by 17% compared to conventional Si source/drain TFETs. Simulation studies such as this one are essential in optimizing devices without costly fabrication and laboratory measurements. Wang et al. [10] proposed a novel Z-gate n-channel MOSFET layout to improve its radiation tolerance. The novel layout can be radiation-hardened with a fixed charge density at a shallow trench isolation of 3.5 $\times \;10^{12}$ cm${}^{-2}$ while offering a small footprint and small gate capacitance when compared to the enclosed gate layout. In [11] Jiang et al. propose a method for phosphorus doping in SiNW using plasma in order to improve the electrical characteristics of the nanowire. The method showed a positive effect on wires with diameters down to 5 nm and improves the I${}_{\mathrm{ON}}$/I${}_{\mathrm{OFF}}$ ratio.

Variability in device performance is another aspect of novel and miniaturized designs which must be addressed if the design is ever to make the leap from theoretical feasibility to industrial relevance. Lorenz et al. [12] examine the statistical and systematic process variations in three-dimensional nanoscale devices such as FinFETs and stacked nanowire transistors. The authors demonstrate the achievements and feasibility of a full simulation of the impact of relevant systematic and stochastic variations on advanced devices and circuits. In [13], Lee et al. used TCAD in order to study the impact of variability on the next generation Si${}_{x}$Ge${}_{1-x}$ channel gate-all-around (GAA) nanowire metal MOSFETs by looking at the effects of random discrete dopants, line edge roughness, and metal gate granularity. After generating 7200 transistor samples and performing 10,000 quantum transport simulations, a statistical analysis is performed, revealing metal gate granularity as the dominant variability source which should be considered.

We would like to take this opportunity to thank all the authors for submitting exceptional and highly relevant research papers to this Special Issue. We would also like to sincerely thank all the reviewers who took precious time to carefully examine and help improve the quality of all submitted papers. Peer review is an essential component of good science and they deserve recognition for the success of this Special Issue. It is our sincere hope that the results provided in this Special Issue prove useful to scientists and engineers who find themselves at the forefront of this rapidly evolving field. Now, more than ever, it is essential to look for solutions to find the next disrupting technologies which will allow for transistor miniaturization well beyond Silicon’s physical limits and the current state-of-the-art.

## References

[1] Klemenschits X., Selberherr S., Filipovic L. (2018). Modeling of Gate Stack Patterning for Advanced Technology Nodes: A Review. Micromachines.

[2] Gnam L., Manstetten P., Hössinger A., Selberherr S., Weinbub J. (2018). Accelerating Flux Calculations Using Sparse Sampling. Micromachines.

[3] Lu N., Jiang W., Wu Q., Geng D., Li L., Liu M. (2018). A Review for Compact Model of Thin-Film Transistors (TFTs). Micromachines.

[4] Hueting R.J. (2018). The Balancing Act in Ferroelectric Transistors: How Hard Can It Be?. Micromachines.

[5] Chang P., Liu X., Liu F., Du G. (2018). Remote Phonon Scattering in Two-Dimensional InSe FETs with High-*κ* Gate Stack. Micromachines.

[6] Cho Y., Lee J., Yu E., Han J.H., Baek M.H., Cho S., Park B.G. (2019). Design and Characterization of Semi-Floating-Gate Synaptic Transistor. Micromachines.

[7] Duan X., Zhang J., Chen J., Zhang T., Zhu J., Lin Z., Hao Y. (2019). High Performance Drain Engineered InGaN Heterostructure Tunnel Field Effect Transistor. Micromachines.

[8] Omura Y. (2019). Empirical and Theoretical Modeling of Low-Frequency Noise Behavior of Ultrathin Silicon-on-Insulator MOSFETs Aiming at Low-Voltage and Low-Energy Regime. Micromachines.

[9] Yang Z., Yang Y., Yu N., Liou J.J. (2018). Improving ESD Protection Robustness Using SiGe Source/Drain Regions in Tunnel FET. Micromachines.

[10] Wang Y., Shan C., Piao W., Li X., Yang J., Cao F., Yu C. (2018). 3D Numerical Simulation of a Z Gate Layout MOSFET for Radiation Tolerance. Micromachines.

[11] Jiang Y., Wang W., Wang Z., Wang J.P. (2019). Incorporation of Phosphorus Impurities in a Silicon Nanowire Transistor with a Diameter of 5 nm. Micromachines.

[12] Lorenz J., Bär E., Barraud S., Brown A., Evanschitzky P., Klüpfel F., Wang L. (2019). Process Variability—Technological Challenge and Design Issue for Nanoscale Devices. Micromachines.

[13] Lee J., Badami O., Carrillo-Nuñez H., Berrada S., Medina-Bailon C., Dutta T., Adamu-Lema F., Georgiev V.P., Asenov A. (2018). Variability Predictions for the Next Technology Generations of n-type Si_*x*_Ge_1−*x*_ Nanowire MOSFETs. Micromachines.

